# A Proof-of-Concept Study on Bioelectric-Based Biosensing for Prostate-Specific Antigen Detection in Serum Samples

**DOI:** 10.3390/bios15080503

**Published:** 2025-08-03

**Authors:** Georgios Giannakos, Sofia Marka, Konstantina Georgoulia, Spyridon Kintzios, Georgia Moschopoulou

**Affiliations:** Laboratory of Cell Technology, Faculty of Biotechnology, Agricultural University of Athens, Iera Odos 75, 118 55 Athens, Greece; g.giannakos@aua.gr (G.G.); smarka@aua.gr (S.M.); georgkon.na@aua.gr (K.G.); skin@aua.gr (S.K.)

**Keywords:** Bioelectric Recognition Assay (BERA), cell-based biosensor, high throughput, impedance, Molecular Identification through Membrane Engineering (MIME), prostate cancer, prostate-specific antigen (PSA)

## Abstract

Prostate cancer is among the most prevalent malignancies in men worldwide, underscoring the need for early and accurate diagnostic tools. This study presents a proof-of-concept and pilot clinical validation of a novel bioelectric impedance-based biosensor for the detection of prostate-specific antigen (PSA) in human serum. The system integrates Molecular Identification through Membrane Engineering (MIME) with the xCELLigence real-time cell analysis platform, employing Vero cells electroinserted with anti-PSA antibodies. Optimization experiments identified 15,000 cells/well as the optimal configuration for impedance response. The biosensor exhibited specific, concentration-dependent changes in impedance upon exposure to PSA standard solutions and demonstrated significant differentiation between PSA-positive and PSA-negative human serum samples relative to the clinical threshold of 4 ng/mL. The biosensor offered rapid results within one minute, unlike standard immunoradiometric assay (IRMA), while showing strong diagnostic agreement. The system’s specificity, sensitivity, and reproducibility support its potential for integration into point-of-care screening workflows. This bioelectric assay represents one of the fastest PSA detection approaches reported to date and offers a promising solution for reducing overdiagnosis while improving clinical decision-making and patient outcomes.

## 1. Introduction

Prostate cancer is among the most frequently diagnosed malignancies in men across the globe. It ranks as the second most common cancer in men worldwide and is the leading cancer diagnosis in several developed nations [1]. Globally, prostate cancer constitutes a significant share of new cancer cases in the male population, with current estimates indicating it accounts for about 15% of all male cancer diagnoses in the United States [2]. Projections suggest that the number of new prostate cancer cases will nearly double over the next twenty years, rising from 1.4 million in 2020 to approximately 2.9 million by 2040 [3]. This growing burden highlights the critical importance of timely diagnosis. Prostate-specific antigen (PSA) testing, a blood-based diagnostic tool, has substantially contributed to earlier detection and improved survival outcomes. In clinical practice, a PSA concentration of 4 ng/mL is commonly used as the diagnostic threshold to distinguish between normal and potentially pathological levels [4].

PSAs are a family of serine proteases referred to as human kallikreins that act as part of the immune system to break down proteins that can cause infections in the reproductive tract of men. Such antigens are synthesized by secretory epithelial cells of the prostate gland and are also present in small amounts in human milk and seminal fluid [5]. The use of PSAs was first approved due to their ability to dissolve semen coagulum in semenogelins, and that same property also made them useful for the medical diagnosis of certain cancers. They are classified as tumor markers, as such biological entities are usually produced in elevated levels by cancerous cells and can be easily detected in patient serum using immunoassays to offer a fast and relatively low-cost validation of prognosis [6]. Early PSA tests are often recommended in hospitals because when used in combination with other diagnostic tools, such as digital rectal exams (DRE), to physically assess the prostate gland, they can help predict cancer diagnosis at less invasive stages, when it is easier to treat patients. Serum levels of PSAs also offer the best way of monitoring the activity of prostate cancer. Results of a prospective screening study showed that the relative risk of death during 7 years of follow-up fell by 20% among men who had received PSA testing at baseline as compared with those who had not been screened [7].

Conventional prostate cancer diagnostics—including immunoradiometric assays (IRMAs), ELISA, chemiluminescent immunoassays, and digital rectal examinations (DREs)—remain clinical mainstays but possess inherent limitations [8]. IRMA and ELISA, while highly sensitive, demand complex infrastructure and extended processing times, hindering rapid clinical decisions and accessibility [9]. Chemiluminescent assays offer automation yet require costly equipment and suffer from assay variability and interference [10]. DRE is subjective, operator-dependent, and has limited sensitivity for early tumors, with its diagnostic utility increasingly questioned [11]. Crucially, PSA screening is associated with significant false positives and overdiagnosis, reportedly detecting only 10–20% of prostate cancers [12,13,14]. These limitations hinder diagnostic precision, particularly at early stages of disease when intervention is most effective.

In response to these challenges, a variety of innovative biosensing technologies have recently been developed for PSA detection [15,16]. Among these, nanoparticle-based aptasensors utilizing various nanomaterials have been developed for both electrochemical and electrochemiluminescent detection of PSA [17,18,19]. Electrochemical biosensors have also been applied to PSA detection, with studies showing correlations between electrochemical readouts and biomarker profiles in patients with negative multiparametric magnetic resonance imaging (mpMRI) for prostate cancer [20]. In a notable example, Yan et al. demonstrated the use of an electrochemical immunosensor incorporating amino-rich vertically ordered mesoporous silica nanochannel films (NH_2_-VMSF), where PSA binding inhibited redox probe diffusion, resulting in signal attenuation [21]. Subramani et al. [22] have also developed a capacitive biosensor based on gold nanoparticles conjugated to Concanavalin A as the binding elements for PSA, with a linear detection range from 10 pM to 100 nM.

Our group previously validated a bioelectric biosensor utilizing mammalian cells engineered with anti-PSA antibodies. It demonstrated enhanced sensitivity and resolution over conventional immunoradiometric assays (IRMAs) for detecting PSA-positive serum samples above the clinical threshold of 4 ng/mL [23]. Building on this foundation, the present study broadens the application of the Bioelectric Recognition Assay (BERA) in conjunction with Molecular Identification through Membrane Engineering (MIME), a technique designed to increase cellular recognition specificity through the electroinsertion of target-directed biomolecules. BERA operates by measuring alterations in the membrane potential of living cells following specific molecular interactions at the cell surface. Through MIME, the biosensor’s selectivity is further refined by introducing thousands of receptor-like entities—such as antibodies or enzymes—directly into the cell membrane [24]. Experimental evidence using fluorescence microscopy has confirmed that these electroinserted receptors are functionally intact and properly oriented, preserving their ability to bind target analytes with high affinity [25]. This strategy represents a robust, non-genetic means of customizing cellular biosensors for rapid, selective, and label-free detection of a wide range of analytes, as already proven in several reported studies [26,27].

The purpose of the present study is to develop and validate a novel diagnostic approach for the early detection of prostate cancer to advance the previously established cell bioelectric biosensor targeting PSA in serum. By integrating Molecular Identification through Membrane Engineering (MIME), a technology based on measuring changes in the membrane potential of cells engineered with anti-PSA antibodies, with real-time impedance measurements using the xCELLigence platform, this study aims to assess the performance of a bioelectric impedance-based biosensor as a rapid, sensitive, and specific tool for PSA detection. This proof-of-concept work seeks to demonstrate the biosensor’s clinical relevance and its potential to be incorporated into point-of-care testing workflows, ultimately contributing to more accurate diagnostics and improved patient outcomes.

## 2. Materials and Methods

### 2.1. Biological Material and Chemicals

The Vero cell line was originally purchased from LGC Promochem (Teddington, UK). Dulbecco’s Modified Eagle’s Medium High Glucose supplemented with L-glutamine, penicillin/streptomycin, and trypsin/EDTA were purchased from Biowest (Nuaillé, France), and the fetal bovine serum from Pan Biotech (Aidenbach, Germany). The PSA antibody and all other reagents were purchased from Sigma-Aldrich (Taufkirchen, Germany). Twenty human whole blood samples, with PSA concentrations ranging from 0 to 26.5 ng/mL, as determined by a standard immunoradiometric assay, were collected from an equal number of patients at the Army Share Fund Hospital of Athens. All experiments were performed using an E-Plate 96 provided by Agilent (Santa Clara, CA, USA).

### 2.2. Cell Modification

The successful electroinsertion of anti-PSA antibodies into the Vero cell membrane has been extensively validated in our previous work, in which the membrane localization and orientation of inserted antibodies were confirmed via immunofluorescence and functional binding assays [25]. According to the established protocol, Vero cells were modified by electroinserting the anti-PSA antibody into their membrane. For 20 min, 2.5 × 10^6^ cells in 40 μL PBS were incubated on ice with 400 μL of antibody concentration at 0.25 μg/mL. After incubation, the mixture was transferred to an electric field of 1800 V/cm, and two square electric pulses were applied according to a procedure described previously [21]. Electroporated Vero cells that were not exposed to the anti-PSA antibody served as controls. Following electroporation, the cells were counted and seeded into the wells of the xCELLigence E-Plate 96 for subsequent impedance measurements.

### 2.3. Real-Time Impedance Monitoring for PSA Detection Using xCELLigence

Electric cell–substrate impedance sensing for PSA detection was conducted using the xCELLigence Real-Time Cell Analyzer Single Plate (RTCA-SP) system (Agilent, Santa Clara, CA, USA). Briefly, 50 µL of pre-warmed cell culture medium was added to all E-plate wells for equilibration, followed by the addition of 50 µL of electroporated anti-PSA antibody or control Vero cells. For cell density optimization, three seeding densities—10,000, 15,000, and 20,000 cells per well in 100 μL of culture medium—were tested. All subsequent experiments were conducted using the optimal density of 15,000 cells per well, based on our findings. The cells were allowed to settle for 30 min at room temperature before the E-plates were reattached onto the xCELLigence analyzer and incubated overnight in a cell culture chamber at 37 °C with 5% CO_2_. The following day, 50 µL of PSA solution at final in-well concentrations (0–10 ng/mL) was added and gently mixed to assess the biosensor’s response to a PSA standard solution. Additionally, the biosensor response to human serum samples was evaluated. A total of 20 human serum samples were analyzed, including those with PSA levels below and above the clinical threshold of 4 ng/mL, by adding 50 μL of each sample to the overnight-seeded cells. The samples were gently mixed by pipetting, and the E-plates were immediately placed into the RTCA-SP system housed within the cell culture incubator. The biosensor response to both the PSA standard solution and human serum samples was continuously monitored for 30 min post-application, with cell index (CI) values being recorded every 15 s. The slope interval was defined using RTCA software 2.1.0 (Agilent, Santa Clara, CA, USA) and used to analyze the cell index (CI) response during the first minute after sample application. Slope values were calculated by applying linear regression to CI measurements recorded between 0 and 60 s. To improve interpretability and facilitate comparison across samples, the resulting slopes (originally in CI/min) were converted to units of 1/h by multiplying by a factor of 60.

### 2.4. Quantification of PSA in Serum via Immunoradiometric Assay (IRMA)

PSA levels in all human serum samples were quantified using a two-site immunoradiometric assay (IRMA) based on non-competitive binding by two monoclonal antibodies targeting distinct epitopes of the PSA molecule (Immunotech S.R.O., Prague, Czech Republic). The system calibration was performed using a series of PSA standards at known concentrations. Additionally, positive control samples containing PSA concentrations were included to validate the calibration curve. All standards and control samples were prepared in bovine serum albumin buffer containing less than 0.1% sodium azide. Whole blood samples were centrifuged at 1300× *g* for 10 min, and 100 μL of the serum was combined with 100 μL of a tracer solution labeled with iodine-125. The mixtures were added to assay tubes pre-coated with the capture monoclonal antibody. Following this, the radiolabeled detection antibody was introduced, and the tubes were incubated with continuous shaking at 300 rpm for 2 h at room temperature. Post-incubation, unbound radioactivity was removed by washing, and the bound radioactivity was measured using a gamma counter (PACKARD Cobra Auto-Gamma, model C5002, GMI, Troy, MI, USA) for 60 s per sample. PSA concentrations were determined by interpolation from the standard curve generated during the calibration step [28].

### 2.5. Study Design and Statistical Analysis

Each sample (control, standard solution, sample) was tested six times, in quadruplicate to ensure reproducibility (n = 24). Statistical differences between groups were assessed using one-way ANOVA in GraphPad Prism version 10.1.2. (GraphPad Software, San Diego, CA, USA), with statistical significance defined at *p* < 0.05. For the analysis of cumulative impedance responses obtained from human serum samples, unpaired t-tests were performed using the same software.

## 3. Results

The real-time impedance-based detection of PSA was carried out using a biosensor developed through the BERA/MIME approach. In this system, Vero cells were membrane-engineered by the electroporation of anti-PSA antibodies, enabling specific interaction with PSA molecules. Upon antigen–antibody binding at the cell membrane, measurable alterations in the cells’ electrical properties occurred. These were monitored using the xCELLigence RTCA system. The biosensor response was expressed as the slope of the CI within the first minute following the addition of PSA standard solutions.

To determine the optimal cell seeding density for PSA detection, three different densities—10,000, 15,000, and 20,000 membrane-engineered cells per well—were evaluated. PSA standard solutions ranging from 0 to 10 ng/mL were applied under each condition, and impedance changes were recorded in real time.

**Figure 1:**
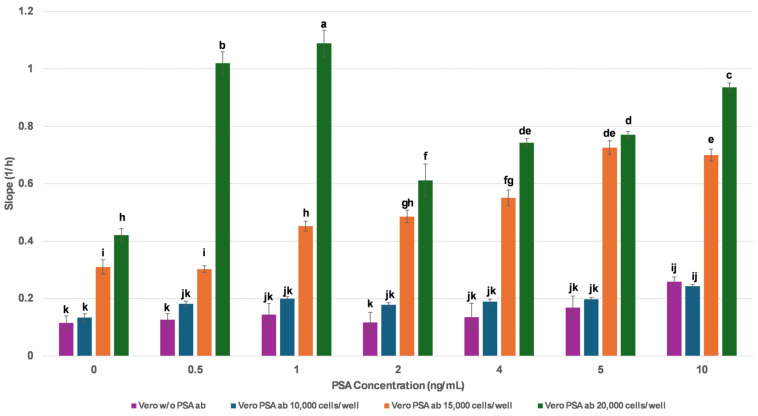
Optimization of membrane-engineered cell density for PSA detection using real-time impedance analysis. Three seeding densities (10,000, 15,000, and 20,000 cells/well) of membrane-engineered Vero cells (Vero_PSAab) were compared, along with non-engineered Vero cells (Vero w/o PSA ab). Data are means ± SEM (n = 24) received from six independent experiments with different batches of cells. Different letters indicate statistically significant differences (*p* < 0.05) based on one-way ANOVA.

As illustrated in Figure 1, non-engineered Vero cells (Vero w/o PSA ab) consistently exhibited low slope values across the entire PSA concentration range (0–10 ng/mL), confirming the absence of non-specific impedance changes and underscoring the necessity of anti-PSA antibody insertion for generating a biospecific signal. In contrast, membrane-engineered Vero cells exhibited measurable responses, as indicated by increased slope values with higher antigen concentrations. The highest absolute slope values were obtained at a cell density of 20,000, indicating strong signal amplitude. However, this configuration showed reduced linearity and variability in the lower PSA range (0–4 ng/mL), which is particularly critical for clinical screening. Conversely, the 10,000-cell condition exhibited the lowest sensitivity and limited dynamic response, suggesting suboptimal performance for PSA detection. Notably, the 15,000-cell configuration demonstrated a clear and consistent dose-dependent trend, especially within the clinically relevant range of 0.5–4 ng/mL. These findings suggest that the 15,000-cell configuration offers a more reliable quantitative output, combining adequate sensitivity with high reproducibility, and enabling more precise discrimination between PSA concentrations. Based on these findings, approx. 15,000 membrane-engineered cells per well were selected as the optimal biosensor configuration, offering the most reliable and analytically suitable response profile for downstream serum-based PSA detection.

**Figure 2:**
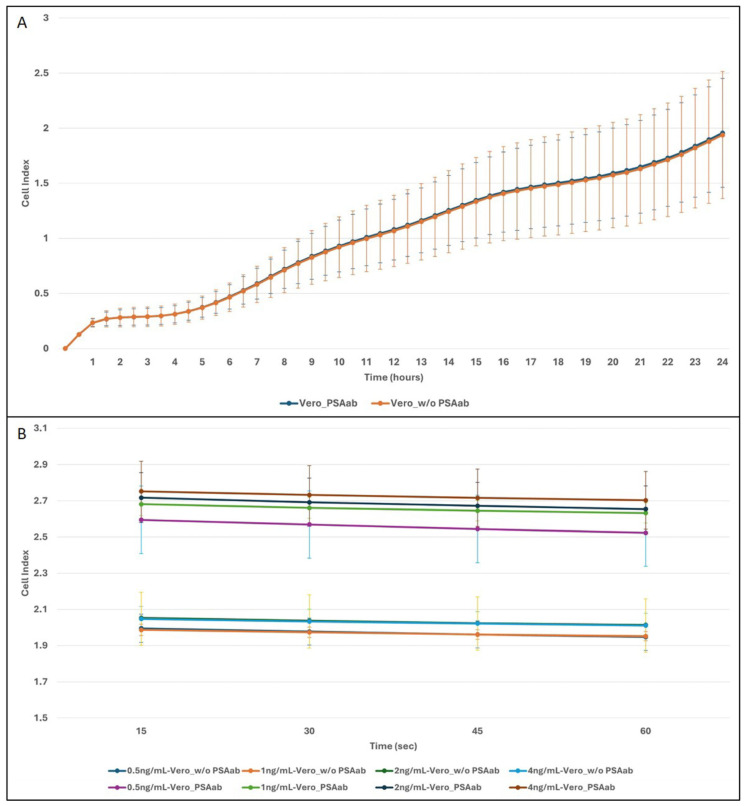
Real-time impedance monitoring of membrane-engineered Vero cells (Vero_PSAab) and non-engineered Vero cells (Vero_w/o PSAab). (**A**) Impedance profiles recorded over 24 h prior to PSA application. (**B**) Impedance measurements during the first minute following the addition of PSA standard solutions (0.5–4 ng/mL). Cell index (CI) values were recorded using the xCELLigence RTCA system.

To assess the specificity and functional performance of the biosensor system, real-time impedance responses of membrane-engineered Vero cells with anti-PSA antibody were compared to electroporated cells without anti-PSA antibody cells following exposure to PSA standard solutions ranging from 0.5 to 4 ng/mL. Prior to PSA application, overnight real-time impedance monitoring was conducted for both cell types to ensure comparable baseline conditions (Figure 2A). The recorded profiles confirmed stable adhesion and growth, providing a consistent starting point for PSA-specific measurements. After PSA application, as shown in Figure 2B, membrane-engineered cells exhibited distinct and concentration-dependent increases in CI within the 1 min measurement window. The CI values correlated positively with PSA concentration, with higher concentrations (2 and 4 ng/mL) producing steeper response curves relative to lower concentrations (0.5 and 1 ng/mL), indicating a robust biosensor response within the clinically relevant PSA range.

In contrast, non-engineered Vero cells showed minimal CI fluctuations across all PSA concentrations, and no concentration-dependent trends were observed. This lack of response in the absence of membrane-inserted antibodies confirms the biosensor’s specificity to PSA-antibody interactions at the cellular membrane level. Notably, the response kinetics in engineered cells were evident within the first minute post-PSA application, supporting the potential of the impedance-based platform for rapid PSA detection.

**Figure 3:**
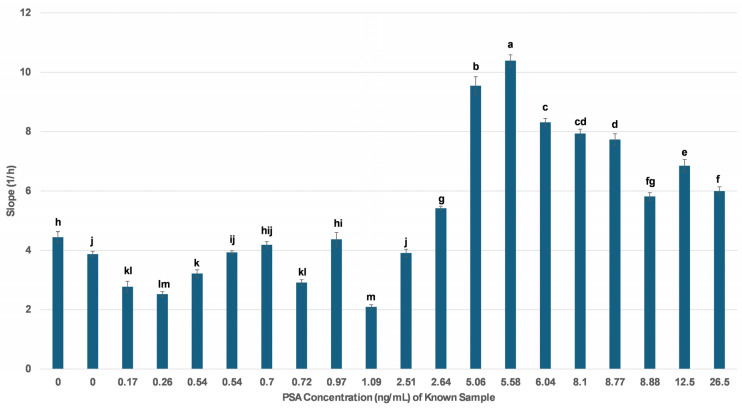
Impedance biosensor response to 20 human serum samples with known PSA concentrations. The slope values (1/h) represent the rate of change in cell index during the first minute after sample application. The data are means ± SEM (n = 24) received from six independent experiments with different batches of cells. Different letters indicate statistically significant differences (*p* < 0.05) based on one-way ANOVA.

To validate the clinical applicability of the impedance-based biosensor, real-time response data were analyzed for 20 human serum samples previously quantified by standard immunoradiometric assay that had known PSA concentrations ranging from 0 to 26.5 ng/mL. As illustrated in Figure 3, the biosensor displayed a quantifiable and reproducible impedance response across the tested range, with slope values (1/h) derived from the first minute post-application showing a general upward trend relative to increasing PSA concentration. Specifically, samples with PSA levels above the clinical threshold of 4 ng/mL exhibited significantly higher slope values compared to those below the threshold, confirming the biosensor’s discriminatory power in distinguishing PSA-positive from PSA-negative serum samples.

The maximum biosensor response was observed at PSA concentrations between 5.06 and 6.04 ng/mL, after which a plateau effect was noted, suggesting a potential saturation of the membrane-bound antibody binding sites at higher PSA levels. In contrast, serum samples with PSA concentrations below 1 ng/mL yielded lower and more variable slope responses, aligning with the expected behavior near the lower limit of detection. Collectively, these findings support the biosensor’s potential as a reliable and sensitive tool for PSA screening in clinical samples.

**Figure 4:**
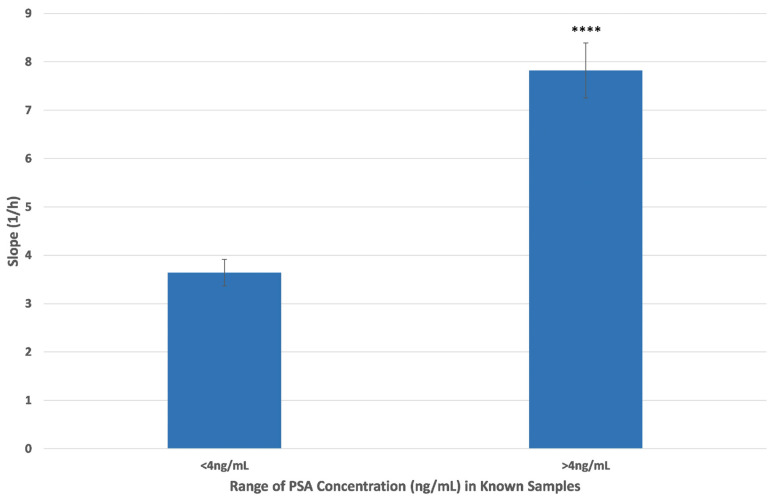
Cumulative impedance biosensor responses to human serum samples with known PSA concentrations above and below 4 ng/mL PSA. The data are means ± SEM (n = 24) received from six independent experiments with different batches of cells. Asterisks indicate statistically significant differences (**** *p* < 0.0001).

To further assess the biosensor’s clinical relevance, a comparative analysis was performed by grouping serum samples into two categories: those with PSA concentrations below 4 ng/mL and those above this clinical threshold. As shown in Figure 4, the biosensor produced significantly different slope values between these groups. Samples with PSA > 4 ng/mL yielded a mean slope of 7.8 ± 0.6, while those with PSA < 4 ng/mL produced a markedly lower average slope of 3.6 ± 0.3, confirming the biosensor’s ability to reliably distinguish between PSA-positive and PSA-negative samples. These results further support the diagnostic utility of the biosensor and its potential to reduce uncertainty in borderline PSA cases near the clinical cutoff.

## 4. Discussion

Recent advances in sensor technologies—including colorimetric, electrochemical, and bioelectronic platforms—have broadened analytical capabilities in health diagnostics and biological quality assessment [29,30,31]. In this context, our study further utilizes a previously established bioelectric biosensing technology for PSA screening based on mammalian cells membrane-engineered with antibodies against PSA [23]. Unlike our earlier work, which focused on cell membrane potential changes, the present investigation measured cell electric impedance, offering a distinct yet complementary biophysical readout of the antigen–antibody interaction.

As clearly shown by the experimental results, bioimpedance can be reliably used to monitor antigen binding on the antibody-bearing cell membrane (in contrast to non-engineered cells) as well as to practically discriminate between negative and positive (>4 ng/mL) blood samples within just one minute of analysis, thus rendering our approach the fastest PSA screening method reported so far. In line with our previous findings, the bioelectric biosensor cannot be used for the qualitative determination of PSAs, especially above 8–10 ng/mL, due to the deviating trend of responses at higher antigen concentrations, an observation frequently attributed to a “hook effect”, i.e., the saturation of the antibody-bearing cell surface with an excessive concentration of the target antigen so that competition between antigens for binding on the membrane antibodies can result in a lower binding efficiency and a lower biosensor response. Such effects are well-documented in immunoassay-based systems [32].

Interestingly, our findings revealed that even non-engineered Vero cells exhibited a modest impedance increase when exposed to PSA-positive human serum. Although significantly lower than the response observed in antibody-engineered cells, this effect may be attributed to nonspecific interactions involving PSA complexes with known protease inhibitors including α_2_-Macroglobulin (α2M) and α_1_-Antichymotrypsin (ACT), which are known to circulate in human serum and can bind to LDL receptor-related protein 1 (LRP1). LRP1 is broadly expressed across various cell types, including kidney-derived lines such as Vero cells, and may mediate nonspecific uptake or membrane interactions [33]. In addition, the PSA’s intrinsic proteolytic activity could potentially modulate the pericellular microenvironment by cleaving extracellular matrix proteins like fibronectin or laminin, subtly influencing cell adhesion and cell–electrode interface properties [34,35]. These nonspecific effects, while minor, may explain the background impedance increases in unmodified cells. Crucially, the electroporation of anti-PSA antibodies into the Vero cell membrane markedly amplified the sensor’s responsiveness, reinforcing the conclusion that the system’s discriminatory performance is primarily governed by antigen-specific binding at the membrane interface.

Cell impedance analysis has been commonly used as an alternative to conventional cytotoxicity assay, for example, in cytopathic effect monitoring following virus infection [36] and exposure to toxic agents [37] as well as the profiling of the bioelectric properties of cancer cells, to monitor their chemoresistivity and sensitivity to chemotherapeutics [38]. Typically, in an electrochemical impedance spectroscopy (EIS)-based immunosensor, the antibody or other capture molecule is immobilized on a conductive electrode surface. Upon the binding of the target antigen, the interfacial impedance at the electrode is altered, enabling detection [39]. Beyond antigen recognition, impedance measurements can also capture receptor-mediated signaling events, as dynamic changes in impedance have been associated with the activation of cell surface receptors following specific biochemical stimuli [40]. To the best of our knowledge, this is the first time that EIS was used to study antigen–antibody binding interactions in a living cell-based system (albeit indirectly). The exact mechanism linking antigen binding on the engineered cell membrane and the observed changes in cell impedance needs to be further studied. One plausible explanation involves alterations in ion fluxes across the plasma membrane—specifically, changes in electrolyte influx and efflux—which may modulate cell-to-substrate conductance, as previously reported for similar systems [25]. Additionally, antigen–antibody binding may trigger cytoskeletal reorganization and changes in cell morphology (e.g., cell shape, adhesion strength, and polarization) that significantly influence cell adhesion and membrane dynamics. Collectively, these alterations can modulate the cell layer’s electrical properties and thus be detected as variations in impedance. In fact, real-time impedance assays like the xCELLigence platform have shown that engaging cell surface receptors leads to measurable impedance shifts that reflect dynamic alterations in cell adhesion and morphology [41,42]. Such findings underscore the ability of impedance-based biosensors to translate complex ligand-induced cellular events into quantifiable electrical signals.

Despite the promising performance of the developed biosensor, several limitations must be acknowledged, particularly those associated with the inherent complexity of human serum as a biological matrix. Serum is rich in proteins such as albumin, immunoglobulins, and lipoproteins, which can potentially interfere with biosensing assays. Albumin, the most abundant serum protein, is known to adsorb nonspecifically to sensor surfaces and may alter electrochemical or bioelectric signals through steric hindrance or by masking target interactions [43,44]. Immunoglobulins, particularly IgG, can also interact nonspecifically with engineered membrane components or the Fc regions of inserted antibodies, potentially affecting binding specificity or triggering unintended signaling responses [45,46]. Moreover, serum components like electrolytes, lipids, and metabolites may influence cell membrane integrity or alter ionic conductivity, thereby impacting impedance measurements [47]. Nonetheless, in our study, these potential matrix interferences did not compromise the biosensor’s functional performance. The biosensor reliably distinguished samples with PSA concentrations above and below the clinical threshold of 4 ng/mL, with results showing strong concordance with those obtained using the standard immunoradiometric assay (IRMA). This suggests that the combination of cell membrane engineering and impedance-based detection provides a level of robustness against nonspecific serum effects, supporting its potential for clinical application. However, it is important to note that the assay is constrained by a relatively short detection window, requiring rapid sample handling and prompt impedance recording to ensure accurate results. In addition, the procedure demands specialized personnel and access to laboratory infrastructure, including cell culture facilities and real-time impedance analysis equipment, which may limit its immediate implementation in decentralized or resource-limited settings.

There is significant potential to enhance cancer treatment by combining biosensing methods that target PSA with personalized medicine strategies, leveraging the vast array of genomics and transcriptomics techniques available in modern healthcare. Given the increasing prevalence of an aging population and the rising demand for healthcare services, future cancer treatment and patient management will particularly benefit from these rapid, cost-effective point-of-care technologies. The standardization of systems for the accurate measurement of PSA is expected to be challenging, and simply detecting the presence of PSA is not enough; it is important to detect differences within healthy ranges, both through disease progression and treatment. Nevertheless, the developments already made are noteworthy in reducing the numbers of tests required, leading to earlier diagnosis and improved patient survival. In this respect, our present report is a further contribution to personalized cancer diagnostics. Due to its working principle, an impedance-based Bioelectric Recognition Assay (BERA) is an analytical process that can be readily integrated with laboratory information systems through appropriate web applications, allowing doctor/patient information at the Point-of-Care, along with the generation of a unique test QR code [27,48]. Eventually, the identification of novel biomarkers other than PSA and the companion development of further biosensing applications will considerably increase the successful score of a sample at the point-of-care use [49,50].

## 5. Conclusions

This paper described our pilot validation of a bioelectric-based biosensor for rapid PSA detection using membrane-engineered mammalian cells, and thus demonstrated its successful development. The sensor effectively distinguished between PSA-negative and PSA-positive human serum samples (above 4 ng/mL) within one minute of analysis, showing high specificity and reproducibility. The impedance response was concentration-dependent in both standard solutions and human serum samples, confirming the system’s functional sensitivity. While high antigen concentrations (>10 ng/mL) affected response consistency—likely due to a hook effect—this did not compromise the sensor’s ability to classify samples as positive or negative. Overall, our findings support the biosensor’s potential as a fast and reliable tool for PSA screening in clinical settings.

## Data Availability

The data presented in this study are available upon request.
